# Organ Doses from Chest Radiographs in Tuberculosis Patients in Canada and Their Uncertainties in Periods from 1930 to 1969

**DOI:** 10.1097/HP.0000000000001171

**Published:** 2020-12-05

**Authors:** David C. Kocher, A. Iulian Apostoaei, Brian A. Thomas, David Borrego, Choonsik Lee, Lydia B. Zablotska

**Affiliations:** 1 Oak Ridge Center for Risk Analysis, Inc., Oak Ridge, TN; 2 Radiation Epidemiology Branch, National Cancer Institute, Bethesda, MD; 3 Department of Epidemiology and Biostatistics, School of Medicine, University of California, San Francisco, San Francisco, CA.

**Keywords:** dose, organ, radiation, medical, x rays, x-ray machines

## Abstract

This paper describes a study to estimate absorbed doses to various organs from film-based chest radiographs and their uncertainties in the periods 1930 to 1948, 1949 to 1955, and 1956 to 1969. Estimated organ doses will be used in new analyses of risks of cancer and other diseases in tuberculosis patients in Canada who had chest fluoroscopic and radiographic examinations in those periods. In this paper, doses to lungs, female breast, active bone marrow, and heart from a single chest radiograph in adults and children of ages 1, 5, 10, and 15 y in the Canadian cohort and their uncertainties are estimated using (1) data on the tube voltage (kV), total filtration (mm Al), tube-current exposure-time product (mA s), and tube output (mR [mA s]^−1^) in each period; (2) assumptions about patient orientation, distance from the source to the skin of a patient, and film size; and (3) new calculations of sex- and age-specific organ dose conversion coefficients (organ doses per dose in air at skin entrance). Variations in estimated doses to each organ across the three periods are less than 20% in adults and up to about 30% at younger ages. Uncertainties in estimated organ doses are about a factor of 2 to 3 in adults and up to a factor of 4 at younger ages and are due mainly to uncertainties in the tube voltage and tube-current exposure-time product.

## INTRODUCTION

Studies of a cohort of approximately 64,000 tuberculosis patients in Canada who had multiple chest fluoroscopic examinations provided estimates of risks of lung cancer, female breast cancer, and heart disease associated with fractionated exposures to ionizing radiation at moderate dose rates of about 0.5 to 0.6 mGy s^−1^ and mean total doses of about 1 to 2 Gy (Miller et al. 1989; Howe 1995; Howe and McLaughlin 1996; Zablotska et al. 2014). Similar analyses of risks of cancer and heart disease associated with chest fluoroscopic examinations were conducted in a smaller cohort of tuberculosis patients in Massachusetts (Boice et al. 1978, 1991; Davis et al. 1987, 1989) and in the two cohorts combined (Tran et al. 2017).

As part of a study to estimate risks of cancer and other diseases in tuberculosis patients in the Canadian fluoroscopy cohort using a new dosimetry system and an extended period of follow-up of disease incidence and mortality, estimates of absorbed doses to various organs from film-based chest radiographs and their uncertainties will be included for the first time. Chest radiographs were used in diagnosing disease and monitoring disease status in patients during treatment for tuberculosis. Although organ doses from a single chest radiograph were about 2 orders of magnitude lower than doses from a single fluoroscopic examination, doses from chest radiographs are potentially important when about 1% of patients in the Canadian cohort had more than 100 radiographic examinations (maximum nearly 1,000), and more than half of patients with chest radiographs did not have any fluoroscopic examinations. The average number of chest radiographs per patient was 21.

This paper describes a study to estimate organ doses from a single chest radiograph and their uncertainties in tuberculosis patients in the Canadian fluoroscopy cohort. Doses to lungs, female breast, active bone marrow, and heart in adults and children of ages 1, 5, 10, and 15 y and their uncertainties are estimated using (1) data on machine parameters in chest radiography in three periods from 1930 to 1969 when exposures of the cohort occurred; (2) assumptions about patient orientation, distance from the source to the skin of a patient, and film size; and (3) new calculations of sex- and age-specific organ doses (Gy) per dose in air (Gy) at skin entrance, referred to as organ dose conversion coefficients (DCCs). Organs in which doses are estimated in this paper are the primary organs in the field of view of an x-ray beam in chest radiography.

Ideally, organ doses from chest radiographs in the Canadian fluoroscopy cohort could be estimated using patient-specific information on machine parameters in each examination. However, the only information on chest radiographs in medical records of patients in the cohort is the total number of examinations, year of admission to a sanatorium, and age at admission. There is no information on machine parameters and other important exposure conditions noted above, and the years in which each patient had chest radiographs can only be inferred from the year at first admission, total number of examinations, and an assumption about the number of examinations per year. In addition, there is no information on chest radiographs prior to or after treatment in tuberculosis sanatoria or radiographic examinations in outpatient settings.

Given the lack of data on chest radiographic procedures in Canadian sanatoria, it is not feasible to estimate doses to individual patients in each period that account for the possible variability in exposure conditions in patients of the same sex and age and from exam to exam in the same patient. Rather, probability distributions of organ doses in each period developed in this paper are intended to represent uncertain *average* doses from a single chest radiograph in males or females of specified ages in the Canadian fluoroscopy cohort.

## MACHINE PARAMETERS

Important parameters in operations of x-ray machines include the peak tube potential (kV), referred to as the tube voltage (ICRU 2005), total filtration (mm Al), product of the tube current and exposure time (mA s), denoted by *P*_I*t*_ (ICRU 2005), and tube output (mR [mA s]^−1^) at a specified distance from the source. The following sections describe available data on machine parameters in chest radiography, as obtained from general scientific literature, and assumptions about those parameters and their uncertainties in three periods when exposures of the Canadian fluoroscopy cohort occurred: 1930 to 1948, 1949 to 1955, and 1956 to 1969. These demarcations in time correspond approximately to changes in National Bureau of Standards (NBS) recommendations on total filtration (NBS 1949, 1955). The first four sections describe data and assumptions about the tube voltage, total filtration, tube-current exposure-time product, and tube output in chest radiography in adults;^4^ assumptions about these parameters and their uncertainties are summarized in Table 1. A concluding section describes assumptions about tube voltages at younger ages.

**Table 1:**
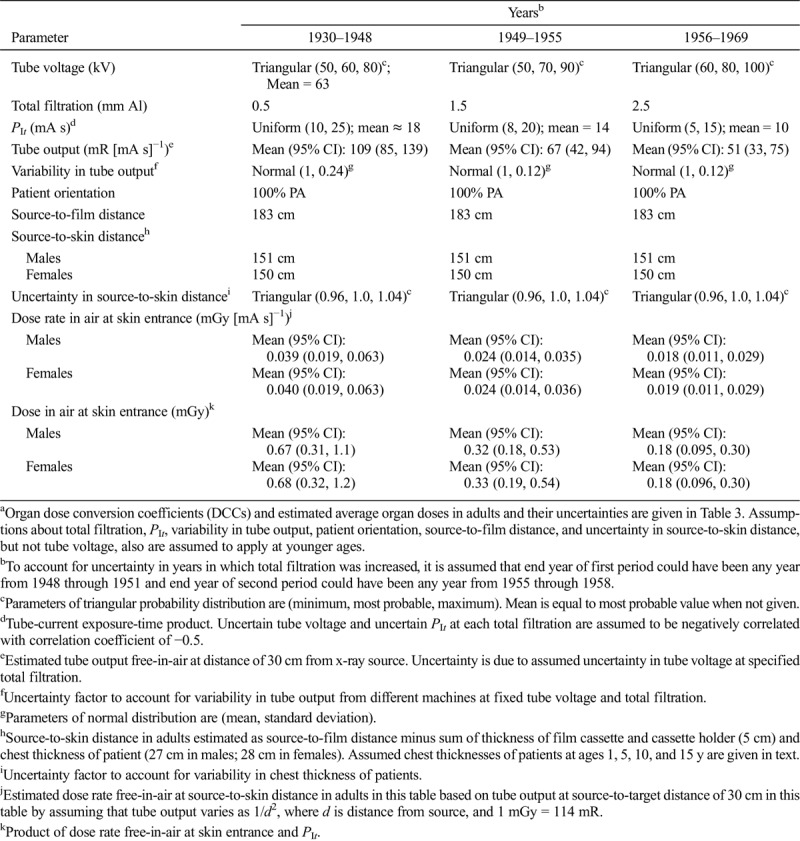
Assumed machine and patient parameters and estimated doses in air at skin entrance from single chest radiograph in adults and their uncertainties in three periods from 1930 to 1969.^a^

Assumptions about patient orientation, source-to-skin distances, film size, and organ DCCs are described in later sections.

### Tube voltage

#### 1930 to 1948

Two manuals from General Electric (GE) X-Ray Corporation in this period (GE 1943, 1945) noted a tube voltage in chest radiography of 60 kV and a range of established tube voltages in operations of GE’s machines of 50 to 80 kV. Another contemporaneous report (Rigler 1946) noted that the tube voltage should rarely be less than 60 kV or greater than 90 kV.

In a recent dose assessment in the United States, Kathren and Shockley (2005) reported that a tube voltage of 80 kV was used at the Hanford site beginning in 1946. Two recent dose reconstructions at the National Cancer Institute used different assumptions about tube voltages in this period. Based on data on practices in the United States in 1964 (Gitlin and Lawrence 1966), Simon (2011) assumed tube voltages of 70, 80, and 90 kV with equal probability, whereas Melo et al. (2016) assumed a lower tube voltage of 53 kV, as reported by Clark (1939, 1949) based on that author’s practices in the United Kingdom.

In this analysis, it is assumed that the tube voltage in chest radiography in adults in this period can be described based on information in GE manuals (GE 1943, 1945), rather than data on practices in the United States in 1964, which may not represent practices prior to 1949, or reported practices in the United Kingdom, which may not represent common practices in North America. The uncertain tube voltage is described by a triangular probability distribution with minimum, most probable, and maximum values at 50, 60, and 80 kV, respectively; the mean of this distribution is 63 kV.

#### 1949 to 1955

Data in Fig. 5.45 of Morgan and Corrigan (1955) suggest that the tube voltage in chest radiography in this period ranged from about 50 kV at a patient’s chest thickness of 18 cm to about 80 kV at a chest thickness of 30 cm. Another contemporaneous report (Ritter et al. 1952) noted that commonly used tube voltages ranged from 40 to 100 kV.

Kathren and Shockley (2005) reported that a tube voltage of 80 kV was used at the Hanford site in 1959. Since the same tube voltage was used at that site beginning in 1946, as noted above, a tube voltage of 80 kV presumably was used in this period. As in the earlier period, Simon (2011) assumed tube voltages of 70, 80, and 90 kV with equal probability based on data in the United States from Gitlin and Lawrence (1966), and Melo et al. (2016) assumed a lower tube voltage of 53 kV, as reported by Clark (1949, 1956) in the United Kingdom.

In this analysis, it is assumed that the tube voltage in chest radiography in adults tended to be higher in this period than in the period prior to 1949. Based on information summarized above but excluding the assumptions by Simon (2011) and Melo et al. (2016), the uncertain tube voltage is described by a triangular probability distribution with minimum, most probable (mean), and maximum values at 50, 70, and 90 kV, respectively. As discussed later, it also is assumed that the increase in tube voltage in this period was accompanied by an increase in total filtration.

#### 1956 to 1969

A GE manual in this period (GE 1965) reported a typical tube voltage in chest radiography of 80 kV and a range of commonly used tube voltages of 60 to 100 kV. Data reported by Gitlin and Lawrence (1966), the US Department of Health, Education, and Welfare (DHEW) and Food and Drug Administration (US DHEW and US FDA 1973), the National Council on Radiation Protection and Measurements (NCRP 1976), and Kathren and Shockley (2005) also indicate that tube voltages probably were in the range of 60 to 100 kV. As in the earlier periods, Simon (2011) assumed tube voltages of 70, 80, and 90 kV with equal probability based on data in the United States from Gitlin and Lawrence (1966). Melo et al. (2016) assumed lower tube voltages of 53 kV with no added filtration prior to 1960 and 65 kV with an added filtration of 2 mm Al (total filtration of 2.5 mm Al) in the 1960s, as reported by Clark (1956, 1964) in the United Kingdom.

Recommendations on total filtration in NCRP Report 33 (NCRP 1968) suggest that tube voltages in this period could have been as low as 50 kV or less with no added filtration (i.e., at an inherent filtration of about 0.5 mm Al) and in the range of 50 to 70 kV with an added filtration of 1 mm Al (total filtration of 1.5 mm Al). Whether such combinations of tube voltage and filtration were used in chest radiography in adults in this period is unknown.

Based on information in several reports in the United States noted above, it is assumed in this analysis that the uncertain tube voltage in chest radiography in adults in this period is described by a triangular probability distribution with minimum, most probable (mean), and maximum values at 60, 80, and 100 kV, respectively. As discussed in the following section, it is assumed also that the increase in tube voltage in this period was accompanied by an increase in total filtration.

### Total filtration

#### 1930 to 1948

Morgan and Corrigan (1955) reported a typical total filtration in medical radiography in the 1940s of 0.5 mm Al; i.e., only the inherent filtration of an x-ray machine, but no added filtration, was used. An assumption of a total filtration of 0.5 mm Al in this period conforms to a recommendation in NBS Handbook 20 (NBS 1936), which was not superseded until 1949. Melo et al. (2016) also assumed a total filtration of 0.5 mm Al in this period, as reported by Clark (1939, 1949) in the United Kingdom.

Kathren and Shockley (2005) reported that an added filtration of 1 mm Al (total filtration of 1.5 mm Al) was used at the Hanford site beginning in the mid-1940s. However, Shockley et al. (2008) apparently assumed no added filtration at those times. The latter assumption also was used by Simon (2011).

Based on the recommendation in NBS Handbook 20 (NBS 1936) and the report by Morgan and Corrigan (1955) noted above, it is assumed in this analysis that only the inherent filtration of an x-ray machine (i.e., a total filtration of 0.5 mm Al) was used in chest radiography in this period. However, as described later, the latest year in which a total filtration of 0.5 mm Al was used is assumed to be uncertain.

#### 1949 to 1955

NBS Handbook 41 (NBS 1949) recommended an added filtration in radiography of at least 1 mm Al; i.e., a total filtration of at least 1.5 mm Al. A total filtration of 1.5 mm Al was noted in contemporaneous reports by Ritter et al. (1952) and Morgan and Corrigan (1955).

Kathren and Shockley (2005) reported that an added filtration of 1.5 mm Al (total filtration of 2 mm Al) at a tube voltage of 80 kV was used at the Hanford site in 1946 and 1959, which implies that the same filtration was used in this period. However, Shockley et al. (2008) apparently assumed a total filtration of 1.5 mm Al in this period, an assumption that also was used by Simon (2011). Melo et al. (2016) assumed no added filtration at a tube voltage of 53 kV, as reported by Clark (1949, 1956) in the United Kingdom.

Consistent with the recommendation in NBS Handbook 41 (NBS 1949) and reports by Ritter et al. (1952) and Morgan and Corrigan (1955), it is assumed in this analysis that a total filtration of 1.5 mm Al was used in chest radiography in this period. However, as described later, the year in which this total filtration was first used is assumed to be uncertain.

#### 1956 to 1969

NBS Handbooks 60 (NBS 1955) and 76 (NBS 1961) recommended an added filtration in radiography of at least 2 mm Al; i.e., a total filtration of at least 2.5 mm Al. As noted previously, recommendations in NCRP Report 33 (NCRP 1968) suggest that only an inherent filtration of 0.5 mm Al could have been used at tube voltages of 50 kV or less, and a total filtration of 1.5 mm Al could have been used at tube voltages of 50 to 70 kV.

Kathren and Shockley (2005) reported that an added filtration of 1.5 mm Al (total filtration of 2 mm Al) was used at the Hanford site in 1959. Shockley et al. (2008) noted, erroneously, that NBS Handbook 60 (NBS 1955) recommended a total filtration of 2 mm Al; as noted above, Handbook 60 recommended a total filtration of at least 2.5 mm Al. Simon (2011) assumed a total filtration in this period of 2.5 mm Al. Melo et al. (2016) assumed total filtrations of 0.5 mm Al at a tube voltage of 53 kV prior to 1960 and 2.5 mm Al at a tube voltage of 65 kV in the 1960s, as reported by Clark (1956, 1964) in the United Kingdom.

In this analysis, it is assumed that a total filtration of 2.5 mm Al was used in chest radiography in this period. This assumption is consistent with recommendations in NBS Handbooks 60 (NBS 1955) and 76 (NBS 1961) and the assumptions of a most likely tube voltage of 80 kV and range of tube voltages of 60 to 100 kV in this period. However, as described below, the year in which this total filtration was first used is assumed to be uncertain.

#### Uncertainty in years of increases in total filtration

Assumptions in this analysis that the total filtration was increased beginning in 1949 and again beginning in 1956 were based on the years of publication of new recommendations by NBS (1949, 1955). Also this analysis accounts for an uncertainty in the years in which the total filtration was increased.

It is assumed that implementation of NBS recommendations on increases in total filtration could have been delayed by up to 3 y; i.e., a total filtration of 0.5 mm Al could have been used until 1952, and a total filtration of 1.5 mm Al could have been used until 1959. To represent this uncertainty, equal weight is given to the two assumptions about total filtration in the years 1949 to 1951 or 1956 to 1958. For example, in estimating organ doses in 1949, 1950, and 1951, equal weight is given to assumptions of a total filtration of 0.5 or 1.5 mm Al. With each assumption about total filtration in the years 1949 to 1951 or 1956 to 1958, associated assumptions about the tube voltage, tube-current exposure-time product, tube output, and their uncertainties are used in estimating organ doses.

As discussed later, the assumed uncertainties in the years in which the total filtration was increased had little effect on estimated organ doses from chest radiographs at any age.

### Tube-current exposure-time product

Although the tube current and exposure time were selected separately in chest radiography in the periods of interest (e.g., GE 1945, 1965), organ doses are proportional to the product of the tube current and exposure time, a parameter denoted by *P*_I*t*_ (ICRU 2005).

#### 1930 to 1948

GE manuals in this period (GE 1943, 1945) noted that *P*_I*t*_ in safe operations of x-ray machines ranged from 10 to 25 mA s. Data on exposure time and x-ray transformer capacity (mA) in a contemporaneous report by Rigler (1946) suggest a *P*_I*t*_ in chest radiography of at least 10 mA s. Campbell (1947) reported that *P*_I*t*_ at one facility in the United Kingdom ranged from 6 to 36 mA s, depending on a patient’s weight, at a tube voltage of 55 kV.

Kathren and Shockley (2005) reported that a *P*_I*t*_ of 25 mA s at a tube voltage of 80 kV was used at the Hanford site in 1946. Melo et al. (2016) assumed *P*_I*t*_s of 20 mA s from 1930 to 1939 and 25 mA s from 1940 to 1949, both at a tube voltage of 53 kV, as reported by Clark (1939, 1949) in the United Kingdom.

In this analysis, it is assumed that *P*_I*t*_ in chest radiography in this period ranged from 10 to 25 mA s. The uncertain *P*_I*t*_ is described by a uniform probability distribution with minimum and maximum values at 10 and 25 mA s, respectively, and mean of about 18 mA s.

#### 1949 to 1955

Data in Fig. 5.45 of Morgan and Corrigan (1955) and data on practices at the Hanford site in 1946 and 1959 (Kathren and Shockley 2005) suggest a *P*_I*t*_ in this period of about 10 mA s when no grid was used or 30 mA s when a Bucky-Potter grid was used.^5^ Ritter et al. (1952) reported that a *P*_I*t*_ of 24 mA s at a tube voltage of 50 kV and 1.5 mm Al total filtration with no grid was used in chest radiography at one facility. Melo et al. (2016) assumed a *P*_I*t*_ of 25 mA s at a tube voltage of 53 kV with no grid, as reported by Clark (1949, 1956) in the United Kingdom.

Data summarized above are insufficient to make a judgment about a credible range of *P*_I*t*_s in this period. However, data from the earlier and later periods suggest that *P*_I*t*_ tended to decrease with increases in tube voltage and total filtration.^6^ Based on assumptions about uncertainties in *P*_I*t*_ in the period 1930 to 1949 discussed above and the period 1956 to 1969 discussed below and an assumption that a grid was not used in chest radiography in Canadian sanatoria, it is assumed in this analysis that the uncertain *P*_I*t*_ in this period is described by a uniform probability distribution with minimum and maximum values at 8 and 20 mA s, respectively, and mean of 14 mA s.

#### 1956 to 1969

A GE manual in this period (GE 1965) noted that *P*_I*t*_ at a tube voltage of 80 kV increased from about 7 mA s at a patient’s chest thickness of 18 to 22 cm to 10 mA s at a thickness of 23 to 25 cm and about 13 mA s at a thickness of 26 to 29 cm. The GE manual also noted that *P*_I*t*_ at a tube voltage of 80 kV could be in the range of about 5 to 15 mA s, depending on the chest thickness. At a thickness of 22 cm, the recommended *P*_I*t*_ was 5 mA s.

Kathren and Shockley (2005) reported that a *P*_I*t*_ of 10 mA s at a tube voltage of 80 kV was used at the Hanford site in this period. Melo et al. (2016) assumed *P*_I*t*_s of 25 mA s at a tube voltage of 53 kV prior to 1960 and 8 mA s at a tube voltage of 65 kV in the 1960s, as reported by Clark (1956, 1964) in the United Kingdom.

In this analysis, it is assumed that *P*_I*t*_ in chest radiography in this period ranged from 5 to 15 mA s. The uncertain *P*_I*t*_ is described by a uniform probability distribution with minimum and maximum values at 5 and 15 mA s, respectively, and mean of 10 mA s.

### Relationship between tube voltage, total filtration, and tube-current exposure-time product

Previous discussions noted tendencies for the tube voltage to increase with increases in total filtration and for the tube-current exposure-time product, *P*_I*t*_, to decrease with increases in tube voltage and total filtration.^7^ In this analysis, these tendencies are accounted for by assuming that the uncertain tube voltage and uncertain *P*_I*t*_ at each total filtration are negatively correlated with a correlation coefficient of −0.5.

### Tube output

The tube output from x-ray machines, defined as the exposure in air (mR) with no patient present (exposure free-in-air) per mA s, is estimated based on measurements. Tube output depends on the tube voltage, total filtration, and distance from the source to the target; the dependence on source-to-target distance (*d*) is approximately 1/*d*^2^. In estimating organ doses, the relevant source-to-target distance is the distance from the source to the skin of a patient.

Data on tube output vs. tube voltage and total filtration at specified source-to-target distances in periods up to 1970 were reported in NBS Handbooks 41, 60, and 76 (NBS 1949, 1955, 1961), Ritter et al. (1952), Morgan and Corrigan (1955), Gitlin and Lawrence (1966), NCRP Report 33 (NCRP 1968), the Radiological Health Handbook (US DHEW 1970), US DHEW and FDA (1973), Kathren and Shockley (2005), and Shockley et al. (2008). Estimates of tube output at the same tube voltage and total filtration in those reports agree within about 25% or less.

Estimates of tube output over a wide range of tube voltages and total filtrations reported by Gitlin and Lawrence (1966) appear to be appropriate for use in estimating organ doses from chest radiographs, especially in the period after about 1955. Compared with tube outputs in Gitlin and Lawrence (1966), estimates at a total filtration of 2.5 mm Al in NBS handbooks from that period (NBS 1955, 1961) are essentially the same at tube voltages of 50 and 60 kV and are 8 to 23% higher at tube voltages of 70 to 100 kV. The earliest estimates in NBS Handbook 41 (NBS 1949) are higher than tube outputs in Gitlin and Lawrence (1966) at all tube voltages; the difference is 6% or less at tube voltages of 50 and 60 kV, increasing to 13 to 25% at tube voltages of 70 to 100 kV.

In this analysis, it is assumed that tube outputs in all periods are described by estimates in Chapter 6, Table 2, of Gitlin and Lawrence (1966), which were based on an analysis of more than 1,000 measurements on nondental x-ray machines in the United States. At a standard source-to-target distance of 30 cm, that table gives estimates of tube output (mR [mA s]^−1^) at tube voltages of 45 to 100 kV and total filtrations of 0.5 to 4.5 mm Al.^8^ Exposure free-in-air in mR is converted to absorbed dose in air using the relationship 1 mGy = 114 mR (Turner 2007).

**Table 2:**
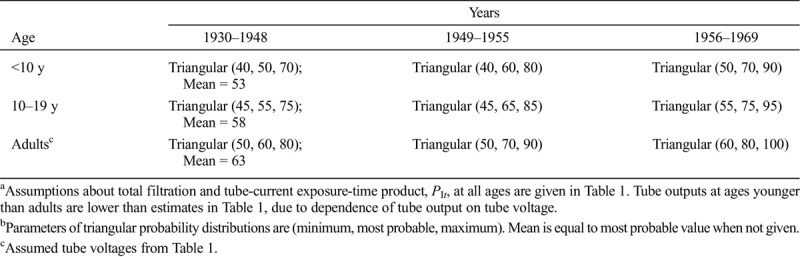
Assumed dependence of tube voltage (kV) from single chest radiograph and its uncertainty on patient’s age in three periods from 1930 to 1969.^a,b^

Based on measurements on 14 x-ray machines, Ritter et al. (1952) reported that variations in tube output at the same tube voltage and total filtration were as great as ±20% of average values. By assuming that those variations applied to machines with added filtration, the uncertainty in tube outputs in the periods 1949 to 1955 and 1956 to 1969 is assumed to be described by a multiplicative factor in the form of a normal distribution with a 90% confidence interval (CI) of (0.8, 1.2) and standard deviation of 0.12.

Data reported by Martin (1947) and discussed by Ritter et al. (1952) indicate that the variability in tube output in machines with no added filtration was about twice the variability in machines with added filtration. Based on that information, the uncertainty in tube output in the period 1930 to 1948 is assumed to be described by a multiplicative factor in the form of a normal distribution with a 90% CI of (0.6, 1.4) and standard deviation of 0.24.

The normal probability distributions to describe uncertainties in tube output are assumed to account for differences in estimates of tube output at the same tube voltage and total filtration in the various reports identified above.

Shockley et al. (2008) noted that an unknown tube output, referred to therein as the beam intensity (*I*), at tube voltage kV could be estimated using the empirical relationship


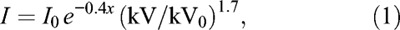


where *I*_0_ is a known tube output at tube voltage kV_0_, and *x* is the thickness of an added filter in mm Al. In later sections, this relationship is used in comparing estimates of organ doses or doses in air at skin entrance from this analysis with estimates from other studies.

### Machine parameters at ages younger than adults

In chest radiography, it is important to obtain images on film of similar quality (exposure and contrast) regardless of a patient’s chest thickness. Since chest thicknesses in children generally are less than in adults, machine parameters in chest radiography in adults should be adjusted at younger ages to obtain about the same image quality at all ages.

Data in GE manuals (GE 1943, 1945, 1965) and Fig. 5.45 of Morgan and Corrigan (1955) suggest that the usual approach to obtaining images of similar quality in chest radiography at all ages was to reduce the tube voltage at ages younger than adults. Reductions in tube voltage, and the resulting decreases in x-ray energies, would compensate for the decreased attenuation of incident x rays at a fixed tube voltage with decreasing chest thickness and the resulting increases in exposure of film and decreases in image contrast.

In this analysis, it is assumed that tube voltages in chest radiography were the same at ages 10 and 15 y and the same, but lower, at ages 1 and 5 y. These assumptions were based on calculations described later that indicated that doses to female breast in chest radiography, and therefore exposures of film at fixed machine parameters, were about the same at ages 10 and 15 y and at ages 1 and 5 y.

Data in GE manuals (GE 1943, 1945, 1965) and Fig. 5.45 of Morgan and Corrigan (1955) suggest that tube voltages in chest radiography were reduced by about 5 kV at ages 10 and 15 y and about 10 kV at ages 1 and 5 y compared with tube voltages in chest radiography in adults. In this analysis, triangular probability distributions in Table 2 are assumed to describe the dependence of the uncertain tube voltage on age in each period.

With the assumed reductions in tube voltage at younger ages, it is assumed that the total filtrations and tube-current exposure-time products, *P*_I*t*_ (mA s), in Table 1 also were used at younger ages; i.e., it is assumed that only the tube voltage was adjusted at younger ages. Estimated tube outputs also are reduced at younger ages as a consequence of the assumed dependence of tube voltage on age and the dependence of tube output on tube voltage.

## OTHER PARAMETERS

### Patient orientation

In this analysis, a posterior-anterior (PA) patient orientation in chest radiography is assumed in all periods. This orientation, which is consistent with general practices in tuberculosis screening and diagnostic procedures, was reported, for example, by GE (1943, 1945, 1965), Rigler (1946), Morgan and Corrigan (1955), Gitlin and Lawrence (1966), and US DHEW and FDA (1973).

Clark (1939) reported that an anterior-posterior (AP) orientation in prone position was used in chest radiography in the United Kingdom when a patient was too sick to assume the preferred PA orientation while standing. Since the fraction of all chest radiographs in AP orientation in tuberculosis patients in Canada presumably was very small, an uncertainty in patient orientation is not accounted for in this analysis.

### Source-to-skin distance

The distance from an x-ray source to the skin of a patient in chest radiography is estimated as the source-to-film distance minus the sum of the thickness of a film cassette and cassette holder and chest thickness of a patient.

The standard source-to-film distance in the United States in all periods of interest was 183 cm. This distance was reported, for example, by GE (1943, 1945, 1965), Rigler (1946), Ritter et al. (1952), Morgan and Corrigan (1955), and Kathren and Shockley (2005). In the United Kingdom, a source-to-film distance of 152 cm was reported by Clark (1939, 1949, 1956, 1964). In this analysis, the source-to-film distance is assumed to be 183 cm in all periods.

The thickness of a film cassette and cassette holder in all periods is assumed to be 5 cm, as reported by Kereiakes and Rosenstein (1980) and the International Commission on Radiological Protection (ICRP 1982). The uncertainty in this thickness is assumed to be negligible.

#### Source-to-skin distances in adults

Chest thicknesses of the hybrid anthropomorphic phantoms in which organ DCCs in adults used in this analysis were calculated (Borrego et al. 2019) are about 27 cm in males and 28 cm in females. Chest thickness varies from patient to patient and, in any patient, is variable over the area of an incident x-ray beam. Based on data in Fig. 5.45 of Morgan and Corrigan (1955), chest thicknesses in adults are assumed to vary by up to ±6 cm from thicknesses in the adult phantoms. This assumption represents interindividual variability and, thus, should overestimate the uncertainty in average chest thicknesses in adults.

Based on the assumed source-to-film distance, thickness of a film cassette and cassette holder, and chest thicknesses, the mean source-to-skin distance is assumed to be 151 cm in adult males and 150 cm in adult females. The assumed variability in chest thicknesses of ±6 cm results in an uncertainty of ±4% in mean source-to-skin distances in adults. This uncertainty is described by a multiplicative factor in the form of a triangular probability distribution with minimum, most probable, and maximum values at 0.96, 1.0, and 1.04, respectively. As noted above, this uncertainty factor should overestimate the uncertainty in mean source-to-skin distances in adults. However, the extent of overestimation should have a negligible effect on the uncertainty in an estimated dose rate in air at skin entrance, which is determined almost entirely by the assumed uncertainty in tube voltage and assumed variability in tube output at the same tube voltage and total filtration.

#### Source-to-skin distances at younger ages

Based on the chest thicknesses of the hybrid anthropomorphic phantoms at ages younger than adults (Borrego et al. 2019), the mean source-to-skin distance is assumed to be 154 cm in males and 153 cm in females at age 15 y, 157 cm at age 10 y (both sexes), 160 cm at age 5 y (both sexes), and 162 cm at age 1 y (both sexes). Uncertainties in mean source-to-skin distances at younger ages are assumed to be described by the triangular probability distribution for adults given above.

### Film size

All sources reviewed in this study, including reports by Rigler (1946) in the United States and Clark (1939, 1949, 1956, 1964) in the United Kingdom, noted that a film size of 36 × 43 cm was used in chest radiography in all periods of interest. This film size was assumed in calculating all organ DCCs used in this analysis.

### Organ dose conversion coefficients

Organ DCCs (dose [Gy] to an organ per dose in air [Gy] at skin entrance) for chest radiographs in PA orientation used in this study (Borrego et al. 2019) were calculated using the most recent hybrid anthropomorphic computational phantoms (Geyer et al. 2014).^9^ Organ DCCs were calculated in adults and children of ages 1, 5, 10, and 15 y using sex- and age-specific assumptions about body weights and heights intended to be representative of a mid-20th century Canadian population with tuberculosis (Thiessen 2017). DCCs for lungs, female breast, active bone marrow, and heart are presented in this paper.

For an assumed field size and location of an x-ray beam at the body surface, organ DCCs depend on the tube voltage and total filtration, which determine the energy spectrum of x rays incident on a patient (beam quality); the higher the tube voltage and total filtration, the higher the average energy of incident x rays and, consequently, the higher the DCC for each organ. Organ DCCs also depend, albeit weakly, on the source-to-skin distance, which affects the angular distribution (extent of collimation) of incident x rays. DCCs for the organs considered in this paper, which are in the field of view of an incident beam, increase as the source-to-skin distance increases, due to an increase in the extent of collimation.^10^

Uncertainties in DCCs for lungs, female breast, active bone marrow, and heart at a specified total filtration are assumed to be due entirely to uncertainty in the tube voltage. Uncertainties in DCCs for those organs due to statistical uncertainties in radiation transport calculations are negligible. For example, the statistical uncertainty in calculated DCCs for lungs in adults, defined as one standard deviation divided by the mean, is about 0.05%.^11^

Sensitivity analyses indicated that variations in the vertical positioning of an incident beam by as much as ±2.5 cm from a central position had negligible effects on estimated doses to lungs in adults. The effects of such variations on estimated doses to other organs considered in this analysis and at younger ages are assumed to be negligible. Other calculations indicated that uncertainties in DCCs for the organs considered in this analysis due to uncertainties in average body mass at each age are about 10% or less. This uncertainty is negligible compared with other uncertainties in estimating organ doses.

## ESTIMATED ORGAN DOSES

For an assumed patient orientation, source-to-skin distance, and film size, an organ dose (mGy) is estimated as the product of the tube-current exposure-time product, *P*_I*t*_ (mA s), the tube output (mR [mA s]^−1^) at skin entrance with no patient present at an assumed tube voltage and total filtration, a conversion from exposure free-in-air to absorbed dose (1 mGy = 114 mR), and an organ-specific DCC (Gy Gy^−1^), which depends on a patient’s sex and age.

### Organ doses in adults

Calculated DCCs and estimated organ doses from a single chest radiograph in adults and their uncertainties are given in Table 3.^12^ As noted previously, 95% CIs of estimated organ doses are intended to represent uncertainties in average doses in tuberculosis patients of the same sex and age in the Canadian fluoroscopy cohort.

**Table 3:**
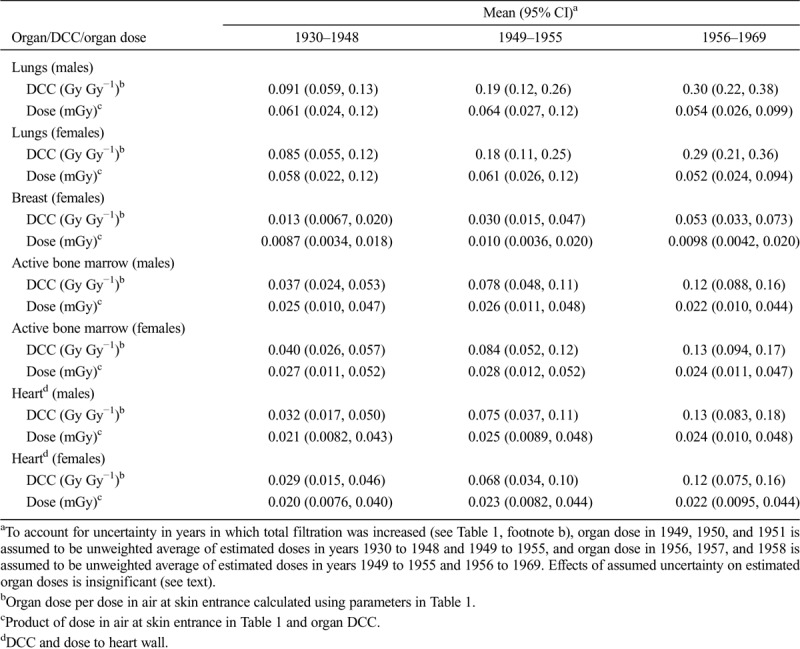
Organ dose conversion coefficients (DCCs) and estimated average organ doses from single chest radiograph in adults in three periods from 1930 to 1969.

Estimated doses to each organ in adults in Table 3 vary by less than 20% across the three periods. These similarities are due mainly to the compensating effects of decreases over time in estimated doses in air at skin entrance (Table 1) and increases in calculated DCCs (Table 3), which are a consequence of assumed increases in the tube voltage and total filtration (i.e., increases in the average x-ray energy) and assumed decreases in the tube-current exposure-time product, *P*_I*t*_ (Table 1). Uncertainties in estimated organ doses in adults, defined as ratios of the bounds of 95% CIs to the means, range from slightly less than a factor of 2 to about a factor of 3 and are due mainly to assumed uncertainties in the tube voltage and *P*_I*t*_.

### Organ doses at younger ages

Calculated DCCs and estimated organ doses from a single chest radiograph at ages younger than adults and their uncertainties are given in Tables 4 to 7. DCCs at younger ages were calculated using the tube voltages in Table 2 and total filtrations in Table 1, and organ doses were estimated using the tube-current exposure-time products, *P*_I*t*_, in Table 1. Tube outputs and dose rates and doses in air at skin entrance at younger ages (not given) are lower than estimates for adults in Table 1. In all periods, estimated doses to each organ at younger ages do not differ significantly from estimates in adults in Table 3.

**Table 4:**
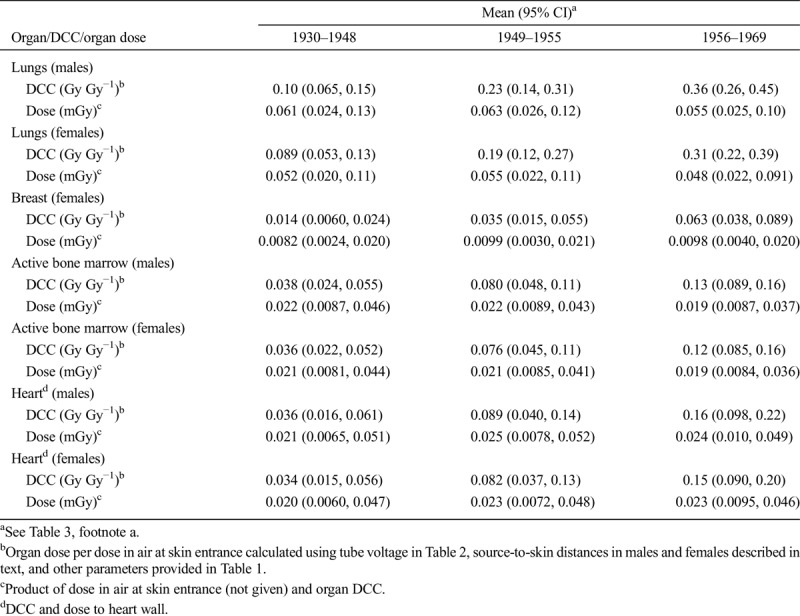
Organ dose conversion coefficients (DCCs) and estimated average organ doses from single chest radiograph at age 15 y in three periods from 1930 to 1959.

**Table 5:**
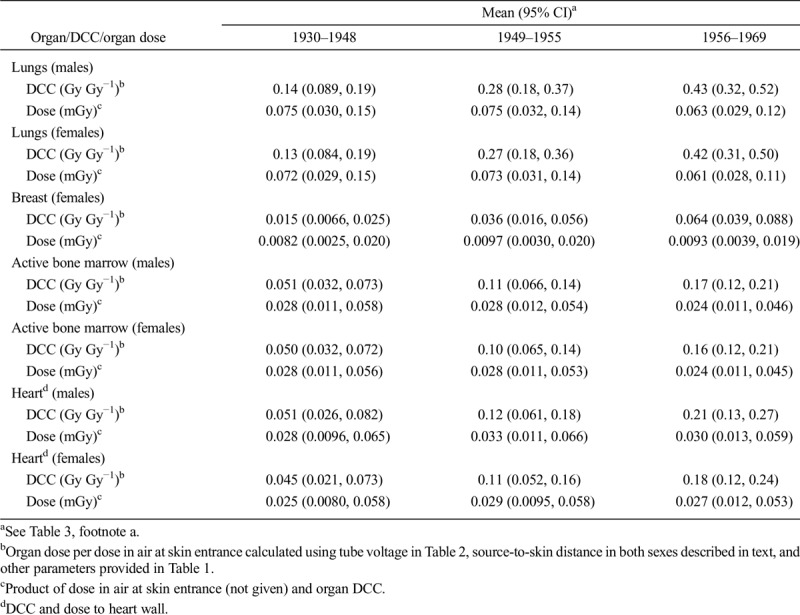
Organ dose conversion coefficients (DCCs) and estimated average organ doses from single chest radiograph at age 10 y in three periods from 1930 to 1969.

**Table 6:**
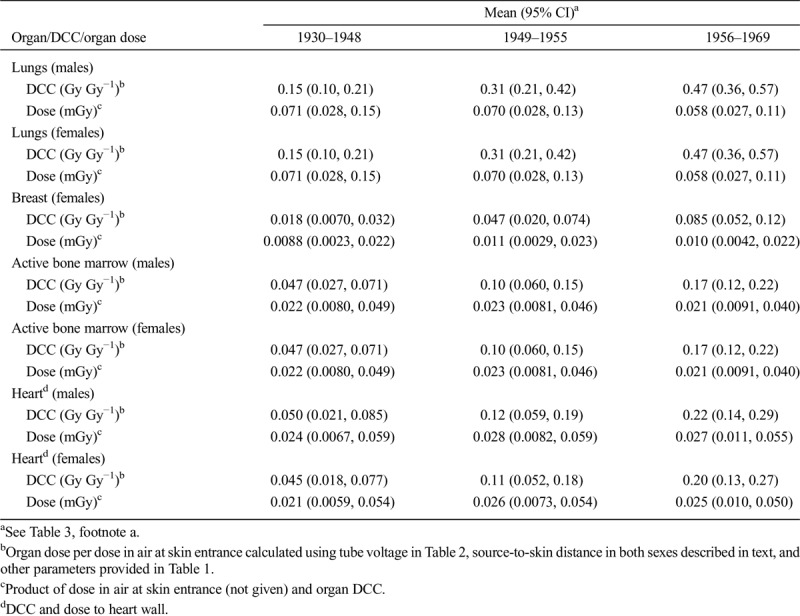
Organ dose conversion coefficients (DCCs) and estimated average organ doses from single chest radiograph at age 5 y in three periods from 1930 to 1969.

**Table 7:**
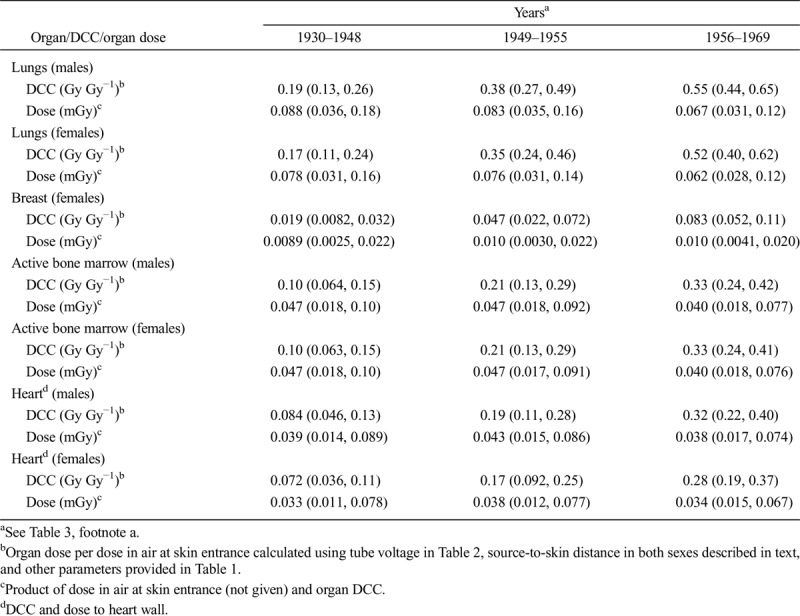
Organ dose conversion coefficients (DCCs) and estimated average organ doses from single chest radiograph at age 1 y in three periods from 1930 to 1969.

The similarities in estimated doses to female breast at all ages and in all periods (Tables 3 to 7) support the assumed reductions in tube voltage with decreasing age in Table 2 and the assumption that the total filtrations and tube-current exposure-time products, *P*_I*t*_, in Table 1 were used at all ages. As noted previously, exposures of film should be about the same in chest radiographs at any age (i.e., regardless of a patient’s chest thickness). Since breast tissue is closest to the film in PA orientation, with no other intervening tissues, doses to female breast in PA orientation are a reasonable representation of exposures of film.

At ages younger than adults, variations in estimated doses to each organ across the three periods are somewhat greater than in adults. The greatest variations of about 30% are seen in estimated doses to lungs in males and females at age 1 y (Table 7). Uncertainties in estimated organ doses (ratios of the bounds of 95% CIs to the means) at younger ages also are somewhat greater than in adults, due to the assumed increases in uncertainty in the tube voltage with decreasing age (Table 2). The greatest uncertainty of nearly a factor of 4 is seen in estimated doses to female breast in the first two periods at age 5 y (Table 6).

### Effect of uncertainties in total filtration

As described previously, an uncertainty in the years in which the total filtration was increased is accounted for in this analysis. However, given the similarities in estimated organ doses at each age across all periods, this uncertainty has little effect on estimated doses in those years. For example, in 1949, 1950, and 1951, when no added filtration or 1 mm Al added filtration are assumed to be equally likely, giving equal weight to estimated doses to lungs in adult males in the periods 1930 to 1948 and 1949 to 1955 in Table 3 yields an estimated dose of 0.063 (0.026, 0.12) mGy. Similarly, the estimated dose to lungs in adult males in 1956, 1957, and 1958, when 1 mm Al or 2 mm Al added filtration are assumed to be equally likely, is 0.059 (0.027, 0.11) mGy. These estimates differ only slightly from the estimated doses at each total filtration in Table 3.

The largest effect of the assumed uncertainty in the years in which the total filtration was increased occurs with the dose to lungs in males at age 1 y in 1956, 1957, and 1958. In that case, giving equal weight to estimated doses in the second and third periods in Table 7 yields an estimated dose of 0.075 (0.032, 0.15) mGy. Again, accounting for this uncertainty does not have a significant effect on the estimated dose.

## COMPARISONS WITH ESTIMATES FROM OTHER STUDIES

### Organ doses

In Table 8, organ doses from a single chest radiograph in PA orientation estimated by Boice et al. (1978) and Kereiakes and Rosenstein (1980) are compared with estimated doses in adults from this analysis.^13^ The same anthropomorphic phantom (Rosenstein 1976) was used to calculate organ DCCs in the previous analyses.

**Table 8:**
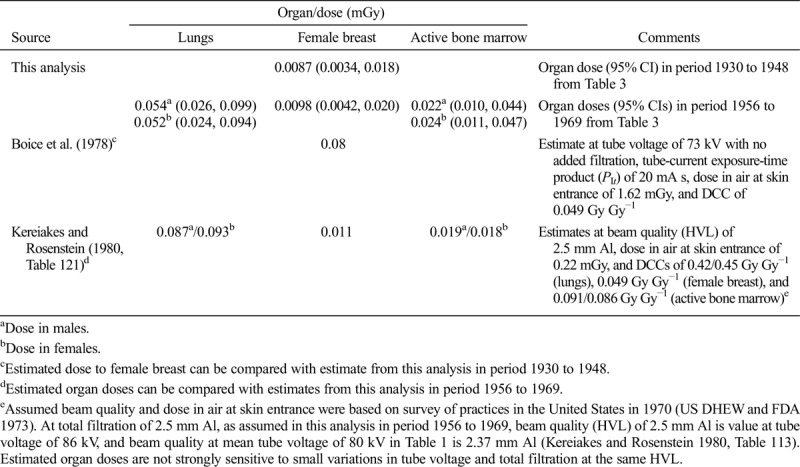
Comparisons of estimated organ doses from single chest radiograph in adults in PA orientation from this analysis with estimates from other studies.

The dose to female breast estimated by Boice et al. (1978) is nearly an order of magnitude higher than the mean dose in adults in the period 1930 to 1949 from this analysis. The higher estimate by Boice et al. (1978) is a consequence of the higher dose in air at skin entrance (1.62 mGy vs. mean of 0.68 mGy in Table 1) and higher DCC (0.049 Gy Gy^−1^ vs. mean of 0.013 Gy Gy^−1^ in Table 3). Using the empirical relationship between the tube output, tube voltage, and added filtration in eqn (1), it is estimated that less than half the difference in doses in air at skin entrance is due to the higher tube voltage assumed by Boice et al. (1978) (73 kV vs. mean of 63 kV in Table 1), which results in a higher tube output at the same total filtration, and higher *P*_I*t*_ (20 mA s vs. mean of 18 mA s in Table 1). The rest of this difference is due to a difference in the assumed tube output at skin entrance (9.25 mR [mA s]^−1^ vs. 5.2 mR [mA s]^−1^ at a tube voltage of 73 kV, total filtration 0.5 mm Al, and source-to-skin distance of 150 cm [Gitlin and Lawrence 1966]). The DCC in female breast of 0.049 Gy Gy^−1^ assumed by Boice et al. (1978) is substantially higher than other calculated DCCs in the same phantom (Kereiakes and Rosenstein 1980, Table 98; Rosenstein 1988, Table 24).^14^ At a tube voltage of 73 kV and total filtration of 0.5 mm Al, the DCC calculated in this analysis is 0.018 Gy Gy^−1^.

In contrast to the comparison with the estimate by Boice et al. (1978), the dose to female breast estimated by Kereiakes and Rosenstein (1980) is consistent with the mean dose and 95% CI in adults in the period 1956 to 1969 from this analysis. This consistency is a consequence of similarities in estimated doses in air at skin entrance (0.22 mGy [Kereiakes and Rosenstein 1980, Table 121] vs. mean of 0.18 mGy in Table 1) and calculated DCCs (e.g., 0.049 Gy Gy^−1^ at a tube voltage of 80 kV and total filtration of 2.5 mm Al [Kereiakes and Rosenstein 1980, Tables 98 and 113] vs. mean of 0.053 Gy Gy^−1^ in Table 3).

Doses to lungs estimated by Kereiakes and Rosenstein (1980) are somewhat higher than mean doses in adults in the period 1956 to 1969 from this analysis, although the previous estimates are encompassed by the 95% CIs of mean doses from this analysis. The higher dose in males estimated by Kereiakes and Rosenstein (1980), for example, is a consequence of the higher dose in air at skin entrance noted above and higher DCC (e.g., 0.39 Gy Gy^−1^ at a tube voltage of 80 kV and total filtration of 2.5 mm Al [Kereiakes and Rosenstein 1980, Tables 98 and 113] vs. mean of 0.30 Gy Gy^−1^ in Table 3). The higher dose in air at skin entrance is due in part to the higher beam quality (half-value layer, HVL)^15^ of 2.5 mm Al vs. 2.37 mm Al at a mean tube voltage of 80 kV and total filtration of 2.5 mm Al (Kereiakes and Rosenstein 1980, Table 113), as assumed in this analysis. The higher the beam quality, the higher the tube output and, consequently, the higher the dose rate in air at skin entrance.

Doses to active bone marrow estimated by Kereiakes and Rosenstein (1980) are consistent with mean doses and 95% CIs in adults from this analysis. The somewhat lower dose in females estimated by Kereiakes and Rosenstein (1980), for example, is a consequence of the lower DCC (0.080 Gy Gy^−1^ at a tube voltage of 80 kV and total filtration of 2.5 mm Al [Kereiakes and Rosenstein 1980, Tables 98 and 113] vs. mean of 0.13 Gy Gy^−1^ in Table 3), which more than compensates for the higher dose in air at skin entrance noted above.

In earlier studies by Laughlin et al. (1957) and Epp et al. (1961) at the same institute, exposures (mR) at locations of active bone marrow per mA s were measured using dosimeters implanted in a physical adult phantom. In Table 9, estimated doses (mGy) to active bone marrow per mA s based on those measurements are compared with estimates based on the mean dose in adult males in the period 1956 to 1969 in Table 3 and mean *P*_I*t*_ of 10 mA s in that period in Table 1. Estimates from this analysis are adjusted to apply at the same tube voltages and total filtrations in the earlier studies using the scaling relationship in eqn (1). Mean doses per mA s in adult males from this analysis are in good agreement with estimates from Epp et al. (1961), but there is less agreement with the earlier estimate from Laughlin et al. (1957).^16^

**Table 9:**
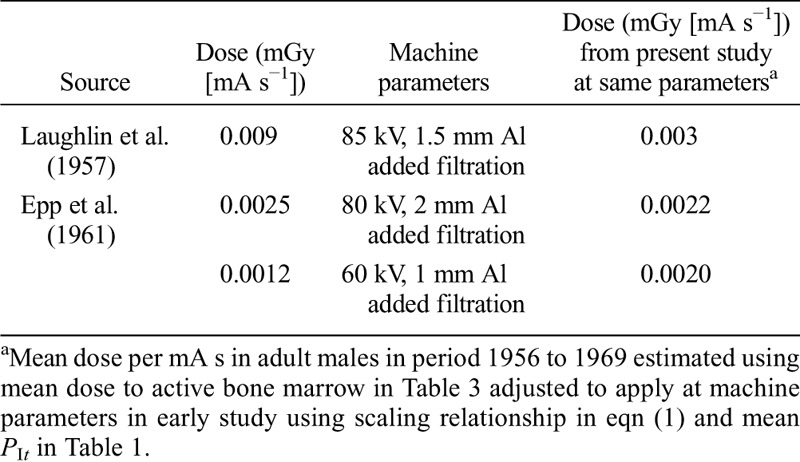
Comparisons of early estimates of doses to active bone marrow per mA s from single chest radiograph with estimates in adult males from this analysis.

### Dose in air at skin entrance

Several early studies reported measurements of exposure (mR) free-in-air at skin entrance at a specified tube voltage, total filtration, and *P*_I*t*_ (mA s) or measurements of exposure per mA s. In Table 10, estimates of absorbed dose in air based on those measurements are compared with estimates based on the mean dose or mean dose per mA s in air at skin entrance in adult males in the period 1949 to 1955 or 1956 to 1969 in Table 1. Estimates from this analysis are adjusted to apply at the same tube voltages and total filtrations in the earlier studies using the scaling relationship in eqn (1) and to apply at the same *P*_I*t*_ when dose is the quantity of interest. As described previously, the uncertainty in estimates from this analysis, which is due to the assumed variability in tube output (mR [mA s]^−1^) at fixed machine parameters, is ±20%. When it is considered that there also is uncertainty in measured exposures in the earlier studies and uncertainty in the scaling relationship in eqn (1), there is generally good agreement between estimates from earlier studies and estimates from this analysis.

**Table 10:**
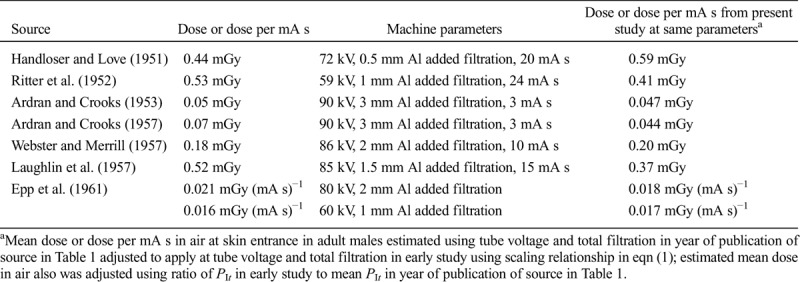
Comparisons of early estimates of doses or doses per mA s in air at skin entrance from single chest radiograph with estimates in adult males from this analysis.

## SUMMARY AND CONCLUSION

This paper has presented results of a study to estimate absorbed doses to lungs, female breast, active bone marrow, and heart from a single chest radiograph in tuberculosis patients in the Canadian fluoroscopy cohort in three periods from 1930 to 1969.

This study emphasized an accounting of uncertainties in sex- and age-specific organ doses from a single chest radiograph in each period. Uncertainties in organ doses were estimated based on assumptions about (1) uncertainties in the tube voltage, tube-current exposure-time product (*P*_I*t*_), and tube output in chest radiography; (2) an uncertainty in the years in which the total filtration in x-ray machines was increased; (3) a negative correlation between the uncertain tube voltage and uncertain *P*_I*t*_ at each total filtration; and (4) an uncertainty in the distance from the source to the skin of a patient. Assumptions about uncertainties in machine parameters were based on data in general scientific literature.

Estimated organ doses in adults and children of various ages and their uncertainties are summarized in Tables 3 to 7. Given the lack of data on chest radiographic procedures in tuberculosis sanatoria in Canada, subjective 95% CIs of uncertain organ doses in each period are intended to represent the state of knowledge of average doses from a single chest radiograph in males or females of specified ages in the Canadian fluoroscopy cohort.

Estimated doses to each organ in adults vary by less than 20% across the three periods. These similarities are due mainly to the compensating effects of decreases over time in estimated doses in air at skin entrance and increases in organ DCCs, which are a consequence of assumed increases in the tube voltage and total filtration and decreases in *P*_I*t*_. At younger ages, variations in estimated doses to each organ across the three periods are somewhat greater and are up to about 30%.

Uncertainties in estimated organ doses in adults are about a factor of 2 to 3. Uncertainties at younger ages are somewhat greater and are up to a factor of 4. Uncertainties in estimated doses are due mainly to assumed uncertainties in the tube voltage and *P*_I*t*_.

In using results in Tables 3 to 7 in analyses to estimate risks of cancer and heart disease associated with radiographic and fluoroscopic procedures in the Canadian fluoroscopy cohort, uncertainties in average organ doses in a particular period will be assumed to be shared among all individuals with chest radiographs in those years; i.e., uncertain doses to all individuals in each period will be assumed to be fully correlated. However, uncertainties in average organ doses will be assumed to be statistically independent across the three periods; i.e., uncertain doses to individuals in different periods will be assumed to be uncorrelated. This approach to accounting for uncertainty is intended to provide unbiased estimates of risk with appropriately broad confidence intervals (e.g., Kwon et al. 2016; Zhang et al. 2017).

## Footnotes

^4^More than 80% of tuberculosis patients in the Canadian fluoroscopy cohort were adults (age ≥20 y) at the time of admission to a sanatorium.

^5^Bucky-Potter grids, when used, were positioned on the opposite side of a patient from an x-ray source to enhance image quality by reducing the quantity of scattered x rays that reached the film or other detector. It is assumed in this analysis that Bucky-Potter grids were not used in chest radiographic examinations in the Canadian fluoroscopy cohort.

^6^An increase in tube voltage increases the average energy of x rays, and an increase in total filtration increases the average energy further by the greater attenuation of lower-energy x rays. As the average x-ray energy increases, a satisfactory image of an exposed patient is obtained at a lower *P*_I*t*_ (mA s).

^7^Simon (2011) assumed the same range of tube voltages in all periods, even though the total filtration was assumed to increase in the periods 1949 to 1954 and 1955 to 1968. Since organ doses to medical personnel were estimated in that analysis using doses measured by film badges, assumptions about *P*_I*t*_ were not needed.

^8^Estimates of tube output vs. tube voltage and total filtration tabulated by Gitlin and Lawrence (1966) were generated using a third-order regression equation to fit the data for nondental x-ray machines. That equation, rather than interpolations of tabulated values, was used in the uncertainty analysis presented in this paper.

^9^Calculated coefficients are organ doses (Gy) per kerma free-in-air (Gy) at skin entrance (Borrego et al. 2019). Absorbed dose free-in-air at skin entrance is assumed to be equal to air kerma at the source-to-skin distances and x-ray energies of interest.

^10^Organ DCCs used in this analysis (Borrego et al. 2019) were calculated without accounting for the thickness of a film cassette and cassette holder of 5 cm. However, when the incident beam is nearly plane-parallel, a discrepancy of about 3% in an assumed source-to-skin distance has a negligible effect on calculated DCCs compared with the effect of the assumed uncertainty in source-to-skin distances on estimates of the tube output at skin entrance.

^11^Statistical uncertainties in calculated DCCs for organs far outside the field of view of an incident x-ray beam (e.g., prostate, ovaries) are as large as 20%.

^12^Mean organ doses and 95% CIs were estimated using Monte Carlo uncertainty propagation techniques with 500 iterations of stratified (Latin hypercube) random sampling.

^13^Organ doses from chest radiographs reported in several studies are not included in comparisons in Table 8. Organ doses at times through 1970 reported by Kathren and Shockley (2005) and Thomas (2011) were excluded on the grounds that doses were estimated assuming an air kerma at skin entrance of 2.0 mGy, which was judged to be a conservative overestimate and is substantially higher than estimated doses in air at skin entrance in any period in Table 1. Comparisons with organ doses reported by Rannikko et al. (1997), Berrington de González and Darby (2004), Hart and Wall (2004), and Hart et al. (2009) may not be meaningful when assumptions about machine parameters or organ DCCs were not reported. Organ doses reported by ICRU (2005) apply at a tube voltage of 120 kV and total filtration of 3 mm Al, which are substantially higher than values of those parameters assumed in this analysis, and organ DCCs were not reported. Organ doses reported by Melo et al. (2016) are the sum of doses in PA and lateral (LAT) orientations, which should greatly overestimate doses in PA orientation (Kereiakes and Rosenstein 1980, Table 121).

^14^Boice et al. (1978) also reported a DCC in female breast of 0.055 Gy Gy^−1^ at an added filtration of 1 mm Al (total filtration of 1.5 mm Al). However, other machine parameters were not reported nor were an estimated exposure or dose in air at skin entrance or dose to female breast. That DCC also is substantially higher than other calculated DCCs in the same phantom.

^15^A half-value layer is the thickness of material required to reduce the tube output by a factor of 2.

^16^The difference in the extent of agreement between estimates from this analysis and estimates from the earlier studies is due in large part to a substantial difference in the assumed distribution of active bone marrow in an adult in the earlier studies. The more realistic distribution assumed by Epp et al. (1961) is closer to the distribution assumed in this analysis.
